# Evaluating peer-supported screening as a hepatitis C case-finding model in prisoners

**DOI:** 10.1186/s12954-019-0313-7

**Published:** 2019-07-05

**Authors:** Desmond Crowley, Ross Murtagh, Walter Cullen, Mary Keevans, Eamon Laird, Tina McHugh, Susan McKiernan, Sarah Jayne Miggin, Eileen O’Connor, Deirdre O’Reilly, Graham Betts-Symonds, Ciara Tobin, Marie Claire Van Hout, John S. Lambert

**Affiliations:** 1 Irish College of General Practitioners, Lincoln Place, Dublin 2, Ireland; 2 School of Medicine, University College Dublin, Dublin, Ireland; 3 Irish Prison Service, Dublin, Ireland; 4 Trinity College Dublin, Dublin, Ireland; 5 Department of Infectious Diseases, Mater Misericordiae University Hospital, Dublin, Ireland; 6 St. James’ Hospital, Dublin, Ireland; 7 Irish Red Cross/Irish Prison Service, Dublin, Ireland; 8 University of Florida, Gainesville, USA; 9 Liverpool John Moores University, Liverpool, UK

**Keywords:** HCV, Hepatitis C virus, Screening, Peer-support, Prison, PWID

## Abstract

**Background:**

Hepatitis C Virus (HCV) infection is endemic in prison populations, and HCV management in prisons is suboptimal. Incarceration is a public health opportunity to target this cohort. Community peer support increases HCV screening and treatment uptake. Prison peer workers have the potential to support the engagement of prisoners with health services and reduce stigma. This study’s primary aim is to evaluate peer-supported screening as a model of active HCV case finding with a secondary aim to describe the HCV cascade among those infected including linkage to care and treatment outcomes.

**Methods:**

An observational study was conducted in a medium-security Irish male prison housing 538 inmates, using a risk-based questionnaire, medical records, peer-supported screening, laboratory-based HCV serology tests and mobile elastography.

**Results:**

A prison peer-supported screening initiative engaged large numbers of prisoners in HCV screening (*n* = 419). The mean age of participants was 32.8 years, 92% were Irish and 33% had a history of injecting drug use. Multiple risk factors for HCV acquisition were identified including needle sharing (16%). On serological testing, 87 (21%) were HCV Ab +ve and 50 (12%) were HCV RNA +ve of whom 80% were fibroscaned (25% showing evidence of liver disease). Eighty-six percent of those with active infection were linked with HCV care, with 33% undergoing or completing treatment. There was a high concordance with HCV disclosure at committal and serological testing (96% for HCV Ab +ve and 89% for HCV Ab −ve).

**Conclusion:**

Peer-supported screening is an effective active HCV case-finding model to find and link prisoners with untreated active HCV infection to HCV care.

## Background

Untreated hepatitis C viral (HCV) infection poses a major public health problem and is endemic in prison populations globally [1, 2]. Prisoners have multiple risk factors for HCV acquisition, the most important being unsafe injecting drug use (IDU), a risk which can exist both in the community and while incarcerated [2–5].

Prison offers an ideal opportunity to target this hard-to-reach group with screening and other healthcare interventions [6–8]. The identification, treatment and prevention of HCV infection in prison populations is a public health priority [4, 9–11] The WHO and other HCV guidelines recommend universal HCV screening for all prisoners [8, 12–16]. Reviews of HCV screening in prisons globally are rare [9, 17], but where available, they show that despite most prisons offering HCV screening, uptake is poor, and standardized protocols for HCV management are the exception [9, 10, 17]. Because most prison sentences are of short duration, HCV linkage to care poses an additional challenge [4, 18, 19].

A number of successful strategies have been shown to increase HCV screening uptake in prisoners. These include the introduction of opt-out rather than an opt-in policy for screening, the use of point of care testing (POC) and dried blood spot testing (DBS) and the targeted screening of at-risk persons on committal (on entry) [9, 10, 17, 20–23]. The uptake of risk-based screening is dependent on prisoners admitting to a history of IDU with its associated stigma, a recognised barrier to both HCV screening and treatment [24].

Recent guidance from the European Centre for Disease Prevention and Control (ECDC) and the European Centre for Monitoring of Drugs and Drug Addiction (EMCDDA) advises that HCV screening be offered to all prisoners and concludes that provider-initiated screening strategies yield a higher uptake than client-initiated strategies [25]. A 2017 systematic review found that HCV screening at prison entry was associated with higher uptake compared to testing during incarceration or pre-release [10]. This review also reported that the use of peer education had a positive impact on the uptake of HIV screening [26].

Of the 600,000 people incarcerated in European prisons at any given time, 3400 are in Irish prisons [18]. Studies on Irish prisoners report high rates of opiate use (50 %), IDU (43%) and HCV infection (13%) [27, 28]. Recent national HCV screening guidelines recommend the screening of all prisoners and re-screening annually with targeted screening if an HCV transmission risk is identified [16]. HCV treatment in Irish prisons is provided by specialist services. Ireland, like other developed countries, has a large proportion of undiagnosed and untreated HCV-infected individuals incarcerated in its prisons [16].

The study site is one of three locations where in-reach hepatology services, through specialist nurses, are provided in the IPS. HCV direct-acting antivirals (DAA) have been available in Ireland since 2014, with initial availability restricted on cases clinical need (including for prisoners) for budgetary reasons. These restrictions were lifted in 2018, and now DAA including 8-week pan-genotypic regimens can be prescribed to HCV-infected prisoners.

Community-based HCV peer workers can increase engagement by people who inject drugs (PWID) with HCV treatment services and reduce associated stigma [29, 30]. Peer-based prison workers have the potential to engage prisoners in healthcare and high levels of support among staff and prisoners further underpin the benefits [31–34].

This study reports on the feasibility and impact of a peer-supported HCV screening and linkage-to-care intervention to increase the numbers of HCV infections detected—in particular new infections, linkage to care, treatment engagement and treatment outcomes in the IPS. While a small number of published studies have reported on the effectiveness of HCV screening initiatives in prisons [10], this study is unique both nationally and internationally in evaluating a peer-supported HCV screening initiative.

## Methods

The IPS partnered with the European Commission Third Health Programme funded ‘HepCare’ Project [35] to enhance screening and primary prevention for populations at risk of HCV infection and specifically implementing an enhanced HCV screening programme at Mountjoy Prison in Dublin Ireland. Ethical approval was obtained from the Mater Ethics Committee as part of the Seek and Treat component of The European Hep Care Project and supported and endorsed by the IPS’s ethics group [36].

### Setting

Mountjoy Prison is a large urban prison which at capacity houses 538 sentenced male prisoners.

### Peer workers in Irish prisons

For many years, the Irish Prison Service (IPS) and Irish Red Cross have trained inmates in all Irish prisons as community-based health volunteers. These prison-based volunteers link with the formal prison health system and act as peer educators to improve prison health and safety. They are volunteers from the prison population on enhance regimes (eligible for defined privileges) and the programme is managed and governed by the Irish Red Cross.

### Intervention development

All Irish Red Cross prisoner volunteers (*n* = 14) were invited to a focus group to discuss their experience of HCV screening and treatment in prison and to provide input into the design and implementation of this study. A draft design of the proposed intervention was completed and presented to a larger implementation group which included prison healthcare and custodial management, prison officers, nurses and doctors and Irish Red Cross staff overseeing the prison volunteer programme.

A researcher-administered questionnaire was developed and piloted by the research team in conjunction with national experts in the area and prisoner groups. The content of the questionnaire was informed by the research tools used in the two previous prison-based prevalence studies and the European ‘HepCare’ project data collection tool [27, 28, 35].

The final intervention design included an awareness and educational session for prison volunteers, educational posters and leaflets as promotional materials, a risk-based questionnaire, provision of HCV screening and result disclosure, referrals for on-site fibroscaning, and linkage to treatment.

### Intervention

The peer-supported screening took place over three, 2-day periods between March 2017 and August 2017. Throughout the study intervention, peer workers accompanied prisoners to the screening sites and promoted the pilot on the landings. This element of the campaign was considered crucial to the engagement of the prisoners in the process. All prisoners were offered BBV screening, but prisoners considered to have severe mental illness undergoing active treatment and prisoners considered to pose a security risk to the research team were excluded from the study (identified by the local medical team).

All study participants were given a patient information leaflet and asked to sign a consent form. No inducements were offered. Study participants were offered blood-borne virus (BBV) testing. Results were given 4 weeks after screening. Results pertaining to prisoners transferred or released were sent on to their relevant medical practitioners. In-reach fibroscaning was available on-site for those found to have HCV infection on screening. Prisoners with untreated chronic HCV infection who remained incarcerated at the study site were referred to in-reach hepatology services for treatment. All clinical data was transferred onto the patients’ electronic medical records.

### Data collection

All prisoners who underwent HCV screening during the peer-supported screening from March 2017 to August 2017 were eligible for inclusion in the study. Data was not collected for all possible participants since the prison population continually changed over this 6-month period.

Data on variables were collected from two sources: the committal interview and the researcher-completed questionnaire. All prisoners routinely complete a nurse committal interview on the day of incarceration which is stored in the prisoners’ medical records in the Prison Health Management System (PHMS). From this medical review, we collected the following variables: age, country of origin, history of drug and alcohol use, presence of visible injecting marks and history of sharing needles.

The questionnaire included questions on age, country of origin, incarceration history, drug use history and HCV risk factors, including history of sharing needles and drug-taking paraphernalia, history of tattooing and the sharing of toothbrushes and razors while incarcerated.

Blood samples were sent to the National Virus Reference Laboratory (NVRL) and tested for HIV, HBV and HCV antibodies. Reflex RNA and genotype testing were performed on all HCV Ab +ve samples. A review of the prisoners’ medical notes was conducted on prisoners testing HCV Ab +ve and RNA negative to determine those with SVR post-treatment and those with spontaneous clearance. This information was cross-checked with the prisoner for accuracy. Twelve months’ follow-up data on linkage to care and treatment outcomes was collected from the participants’ electronic medical records.

### Statistical analyses

All data were anonymised and coded, double-entered and checked. Statistical review of the study was performed by a biomedical statistician (EL), and analysis was conducted using the Statistical Package for Social Sciences (version 23.0; SPSS UK Ltd.; Chersey, UK). Data were assessed for normality and where necessary, data were log-transformed for normalisation purposes. Data within tables are primarily expressed as means (SD) or *n* (%).

## Results

### Demographics

A total of 425 male prisoners consented to participate in the study. Study participants had a mean age of 32.8 years and 92% reported Ireland as their country of origin. The mean age of the first incarceration was 20 years, the mean number of incarcerations was 6 and the mean total time spent incarcerated was 7.7 years. Data from committal interview showed that almost 50% of participants had a past history of drug use. Of those who answered the questions on drug use in the risk questionnaire, 45% had a history of heroin use and 33% a history of IDU. The mean age of first drug use was 15 years and first IDU was 20 years. In terms of risk factors for HCV acquisition (data collected from risk questionnaires), 34.5% gave a history of sharing drug taking equipment (paraphernalia), 15.8% of sharing needles in the community, 17.3% of having had a prison tattoo and 14.2% a non-sterile community tattoo. Small numbers reported sharing a razor or toothbrush in a prison setting (4.2 % and 0.8% respectively). A total of 36.3% reported having a history of methadone treatment, and the mean length of time on treatment was 4.9 years (Table 1).

**Table 1:** Demographics of study participants from Mountjoy Prison (March to August 2017)

Variable	Participant numbers	Value *n* (%)	Mean (SD)
Age^a, b^	425		32.8 (8.9)
Age of first incarceration^a^	364		20.0 (7.1)
Episodes of incarceration^a^	359		5.9 (8.3)
Total time incarcerated (years)^a^	361		7.7 (6.6)
Age of first drug use^a^	281		15.4 (7.7)
Age of first IV use^a^	106		20.1 (5.4)
Previous drug use^b^	409	199 (48.7)	
Shared needles^b^	406	26 (6.4)	
Country of origin (*n* = 425)^b^			
Ireland		389 (91.5)	
West Europe		4 (0.9)	
East Europe		22 (5.2)	
Africa		10 (2.4)	
HCV acquisition risk factors			
History of heroin use	355	161 (45.4)	
History of IV use	341	111 (32.6)	
Shared needle community	342	54 (15.8)	
Shared equipment	344	120 (34.9)	
Shared razor in prison	357	15 (4.2)	
Shared toothbrush in prison	358	3 (0.8)	
Prison tattoo	358	62 (17.3)	
Unsterile tattoo community	346	49 (14.2)	
Opioid substitution treatment			
Methadone treatment history	342	124 (36.3)	
Length of time on methadone maintenance treatment	101		4.9 (6.6)

*SD* standard deviation, *IV* intravenous, *HCV* hepatitis C virus

^a^Values are means (± SD) for continuous variables or *n* (%) for categorical variables

^b^Data from committal interview

### Screening results

**Fig. 1:**
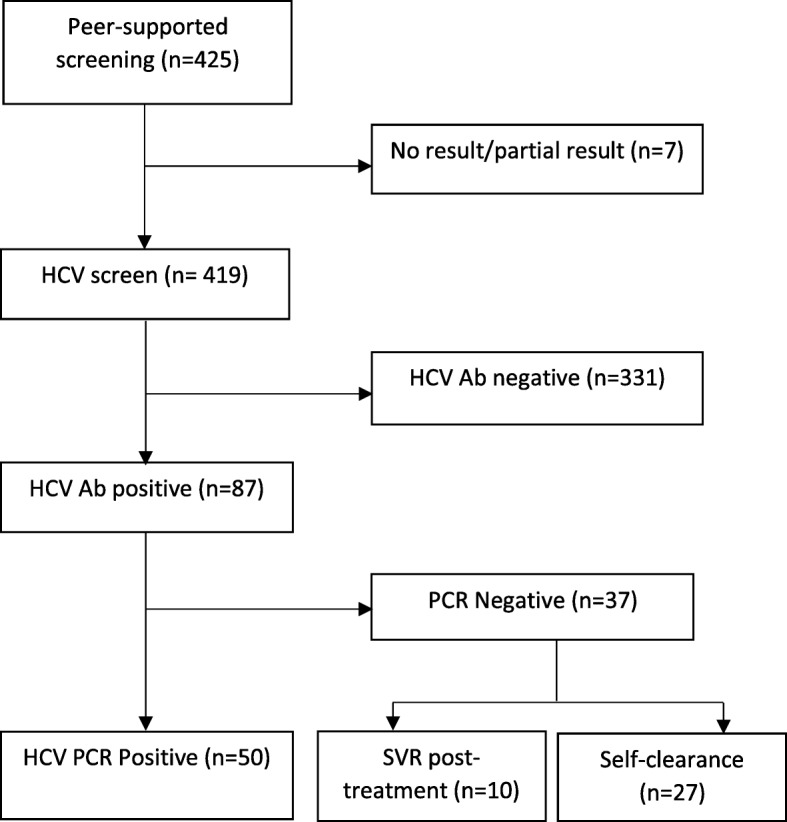
Peer-supported screening outcomes from Mountjoy Prison, Dublin, Ireland (March 2017 to August 2017)

Of the 425 prisoners who consented to participate, 419 had a successful serological HCV result. Eighty-seven (21%) were HCV Ab +ve, 4 (1%) HIV Ab +ve and 3 (< 1%) HBV core Ab +ve. Of those who tested HCV Ab +ve, 37 (43%) were HCV RNA −ve, of whom 27 (31%) had self-cleared and 10 (27%) had SVR. The remaining 50 (57%) showed active HCV infection (HCV RNA +ve) representing 12% of the entire study population (Fig. 1).

**Fig. 2:**
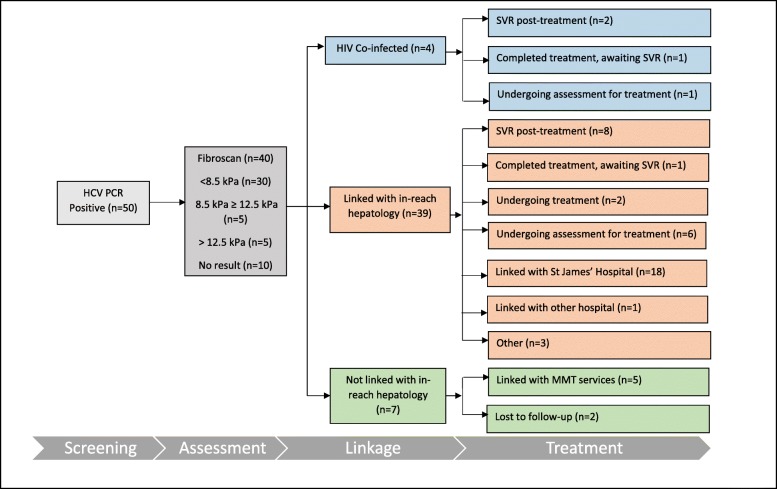
Peer-supported screening—untreated chronic infection outcomes from Mountjoy Prison, Dublin, Ireland (September 2018). HCV hepatitis C virus, PCR polymerase chain reaction, kPa kilopascal, HIV human immunodeficiency virus, SVR sustained virological response, MMT methadone maintenance treatment

### Comparison between self-declared status on committal and serological result on peer-supported screening

The data on self-declared HCV status on committal (collected from the committal interview on the prisoner’s electronic patient record) was grouped into declared HCV Ab +ve on committal, declared HCV Ab −ve on committal and status unknown (never tested or status unknown). Of those screened, 48 (11%) self-declared HCV Ab +ve at committal, and on screening serology, 46 of whom were HCV Ab +ve, showing a concordance of 96%. Of the 171 who declared HCV Ab −ve on committal, 19 (4%) were HCV Ab +ve on serology and eight (2%) were HCV RNA +ve (active infection). This demonstrates a concordance of 89%. Finally, for those unaware of their status at committal, 22 (11%) were HCV Ab +ve and 11 (5%) were HCV RNA + ve. A total of 19 (5% of the study participants screened) new active cases of HCV infection (HCV RNA +ve) were identified through peer-supported screening.

### Linkage, assessment and treatment outcomes

Of the 50 prisoners with active HCV infection, 40 (80%) had in-reach elastography performed. Of this cohort, 30 (75%) had scores < 8.5, five scores of 8.5 > 12.5 (fibrosis) and five scores of ≥ 12.5 (cirrhosis). The outcomes for linkage to care were grouped into three categories: HIV co-infection who were already linked with specialist hospital services (*n* = 4), linked with in-reach hepatology nurse (either already linked or new referral) (*n* = 39) and not linked with either of these services (due to release or inter-prison transfer) (*n* = 7). Treatment outcomes were reported as of September 2018 (> 12 months after the peer-supported screening) and under the following headings: completed treatment with SVR, completed treatment awaiting SVR, undergoing treatment, undergoing assessment for treatment and linked to hospital services or community MMT (the study’s ethical approval did not allow for data to be collected on prisoners after release).

For the HIV co-infected group, three had completed treatment (two achieving SVR and one awaiting an SVR result) and one was undergoing assessment for treatment. Of those linked with in-reach hepatology services, nine had completed treatment (eight achieving SVR and one awaiting an SVR result), two are on treatment and six are under assessment for treatment. Eighteen of this group were referred to the hospital (St. James’), the community location of the specialist hepatology services that provides in-reach to the IPS. Of the final group, five were formally linked to community MMT services and two were lost to follow-up. In summary of the 50 prisoners identified with active HCV infection, 43 were linked to specialist services and of this group, 12 had completed treatment, 10 achieving SVR, two were under treatment and eight were being assessed. Eighteen of this group had a formal direct referral to the hospital setting providing the in-reach hepatology service (Fig. 2).

## Discussion

This unique Irish prison-based study found that peer-supported screening is a feasible active HCV case-finding intervention in a prison setting. It is a convincing example of the benefits of a collaborative prison health intervention using peer-to-peer health promotion and the WHO recommended complete prison approach to planning and implementation [37]. It was successful in testing a large number of prisoners for HCV infection (*n* = 419). It also had the added benefit of testing this cohort for HIV and HBV infection, BBVs with high prevalence in prison populations [2, 38].

Over half of the study population had a history of drug use, with significant numbers having a history of heroin use and IDU. These figures are similar to other Irish and international studies and reflect the over-representation of PWID in prison populations globally due to the ongoing criminalisation of this underserved and marginalised group [2, 27]. This study also found high levels of self-reported known HCV risk behaviours in this prison cohort including IDU, sharing needles and other drug-taking paraphernalia and having a prison or non-sterile community tattoo [2, 5, 39]. Peer-supported screening identified 50 cases (12% of the study population) of active untreated HCV infection of which 19 (5% of the study population) had not been identified at committal. These findings support the public health focus on prisons as key locations to increase HCV diagnosis, linkage to care and treatment [4, 25, 40]. It also supports the ongoing need to increase harm reduction services (opioid substitution treatment and needle and syringe programmes) within prisons to reduce the risks of BBV transmission in closed settings [41, 42].

The use of peer-supported screening as an active HCV case-finding intervention has not been described previously in the literature [25]. This intervention is a provider rather than client-initiated, a factor which is known to improve uptake [25]. It is an intervention that can be used in prison populations who are already incarcerated and not just for those entering prison. Evidence suggests that screening offered within the first 24 h of committal has a better uptake than screening offered later in the custodial sentence or just at pre-release [16, 25]. It is important to remember that efforts to scale up HCV screening and treatment in prisons are a very recent public health intervention and many prisoners globally have been incarcerated since this approach has been more widely adopted. Because of the ongoing risk of HCV transmission during incarceration, updated international guidelines recommend the repeat screening of all prisoners yearly which will require different strategies than those for screening new entrants to prison [9, 16, 25]. Peer-supported screening has the potential to be utilised in these situations.

The use of peers to design, support and implement is intrinsic to this study intervention. Prisoners are identified as a hard-to-reach population, and even in prisons with easier access to healthcare, many still do not engage. There is often a lack of trust between prisoner and staff, and prisoners have identified the fear of stigma as being a key barrier to engagement in the HCV cascade of care [43–45]. The benefits of peer involvement in community HCV care are well documented [30, 46–50]. Peer workers can dispel the myths and fears associated with HCV treatment, reduce stigma, enhance mutual trust, increase social support and increase knowledge and engagement in HCV care [30, 50].

Studies have shown that peer workers have a positive impact on the uptake of HCV services and have high levels of satisfaction among service users and staff [46, 51]. There is further evidence to suggest that engagement in HCV care may be facilitated by the influence of peers who completed treatment. The ETHOS study in Australia reported a very strong positive response to peer workers by staff and service users which led to improved access to services, a more client-friendly treatment environment and increased support to services users with assessment and engagement with HCV treatment [30].

A 2016 systematic review of peer education and support in prison settings found that peer education interventions are effective at reducing risk behaviour, acceptable within the prison environment and have a positive impact on prisoner wellbeing [52]. Peer workers are a credible source of information and have the ability to connect with other prisoners, reduce social stigma and impact positively with a vulnerable patient cohort who is traditionally resistant to professional advice [31]. There are also direct benefits for the peer workers themselves and benefits for the wider prison system including more effective use of resources and the ability to expand the range of prison-based health services available to inmates [51]. This study identified peer workers as an enabler to prisoners engaging in HCV screening and treatment and reducing stigma.

A number of community-based studies have reported on the concordance between perceived HCV status and actual status in PWID [53]. Similar to these studies, this study found high levels of concordance between self-declared and serological HCV status. There was a 96% concordance for those who declared being positive and 89% for those who declared being negative. These findings are contrary to a 2000 Irish study that found self-declared HCV status as unreliable with 37% of those declaring negative being positive on oral swab testing [54]. The difference in findings may be accounted for by the increased numbers attending and the high rates of HCV testing in community drug treatment services in Ireland. The findings from this evaluation suggest that prisoners can be directed into different care pathways based on self-declared status at committal. This may reduce assessment times and improve linkage to care and treatment outcomes an important consideration in prisons where the majority of prisoners serve short sentences [18, 19].

The discordant findings, despite low numbers, are a concern given they represent potential HCV infection risk and re-enforce the need for regular testing and re-testing of prison populations. These findings also highlight the need to improve how we collect HCV data on individuals and populations. HCV-infected people often are unaware of the difference between past infection, chronic infection, active infection, self-clearance, SVR and re-infection. This lack of clarity is also shown in population HCV surveillance data. Historically HCV prevalence data in prison populations was reported as HCV Ab +ve prevalence, some more recent studies have reported on HCV RNA +ve prevalence (current active infection) [1, 2, 55]. As we scale up HCV treatment, it is important that serological markers are matched with clinical data so that we can measure levels of active untreated infection, treated infection and re-infections. Furthermore, it is important that HCV-infected persons are educated on the different phases of HCV infection and their associated blood markers so that they can provide accurate medical information to healthcare staff. Increased accuracy could reduce the need for unnecessary and expensive repeat screening.

This study reports high-levels of linkage to care for prisoners identified as having untreated HCV infection. The presence of the specialist in-reach hepatology nurse facilitated this process with nearly 80% linked with this service. The use of specialist nurses in prisons has previously been identified as a facilitator to HCV screening and treatment in prison settings [23, 43]. Treatment outcomes were impacted by a national decision to curtail DAA access to those with advanced liver disease in June 2017. This restriction was lifted in February 2018. The treatment outcomes reported in this study support previously published findings that prisoners (including those infected with HIV) can be successfully treated for HCV with outcomes similar to or better than other population groups [56, 57]. Many of the HCV-infected participants identified required linkage to hospital-based specialist services on release. This finding underpins the need to support prisoners transitioning back into the community where a range of competing priorities can impact on their ability to link with these services [58, 59]. This transition between prison and community is identified as a high-risk period for PWID and pivotal to HCV treatment uptake, prevention and elimination [19, 60]. This model involved the linking of prisoners not started on treatment to the specialist hospital service that provides in-reach hepatology services to the IPS. It was hoped that this approach might increase uptake in the community since the specialist nurse was common to both locations and would be known to the patient.

The use of HCV serology markers and current fibroscan scores are strengths of this study. The large numbers screened and followed 12 months later is a further strength of this unique study. There are a number of limitations to this study including it being male only and single site, which reduces its generalisability. A further limitation of this study is that it is observational in design and does not have a comparative arm. Comparing the effectiveness of different active HCV case-finding models would increase the utility of its findings, but the implementation of such a study design in a large working prison is difficult. Consent to follow up prisoners on release to the community would have benefitted the study’s findings. Data on the cost-effectiveness of this model is currently underway and will be published at a later date.

## Conclusion

Large numbers of Irish male prisoners having a history of IDU are frequently incarcerated from a young age and have multiple risk factors for HCV acquisition. A peer-supported screening initiative is both a feasible and an acceptable model of active HCV case finding in a prison setting. This model identified 50 cases of untreated active HCV infection of which 19 had not been identified at committal. Prison-based hepatology nurse specialist facilitated the linking of 39 active HCV-infected prisoners to HCV assessment and care. Prisoners can be successfully treated in prison settings, but significant numbers will still require linkage to community HCV treatment services. Supporting prisoners while transitioning to the community is key to optimising HCV management. There is a high concordance between prisoners’ self-declared HCV status and serology status at committal. This finding supports the devolvement of HCV treatment pathways based on self-declared HCV status which could reduce assessment time and linkage to treatment particularly for the large numbers of prisoners who serve short prison sentences. The complexities of prison environments require a planned and coordinated approach to HCV care to optimise outcomes. Incarceration offers an ideal public health opportunity to engage with and support a high-risk group of HCV-infected PWID with prison and community-based health services.

## Data Availability

The datasets used and/or analysed during the current study are available from the corresponding author on reasonable request.

## Abbreviations

+ve: Positive
Ab: Antibody
BBV: Blood-borne virus
DAA: Direct-acting antivirals
DBS: Dried blood spot
ECDC: European Centre for Disease Prevention and Control
EMCDDA: European Centre for Monitoring of Drugs and Drug Addiction
HBV: Hepatitis B virus
HCV: Hepatitis C virus
HIV: Human immunodeficiency virus
IDU: Injecting drug use
IPS: Irish Prison Service
IV: Intravenous
LTFU: Lost to follow-up
MMT: Methadone maintenance treatment
NVRL: National Virus Reference Laboratory
PCR: Polymerase chain reaction
PHMS: Prison Health Management System
POC: Point of care
PWID: People who inject drugs
RNA: Ribonucleic acid
SD: Standard deviation
SPSS: Statistical Package for Social Sciences
SVR: Sustained virological response
−ve: Negative
WHO: World Health Organization
